# Levelling the ground for decolonising the nursing curriculum: A matter of critical consciousness

**DOI:** 10.4102/hsag.v30i0.3207

**Published:** 2025-08-22

**Authors:** Agnes Makhene

**Affiliations:** 1 Department of Nursing, Faculty of Health Sciences, University of Johannesburg, Johannesburg, South Africa

Since 2015–2016, there has been a push in South African higher education to decolonise the university curriculum; however, aside from sporadic efforts by individual educators, not much has emerged. Nurse educators have also not done much in this area. Decolonising the curriculum has proven to be an elusive phenomenon, as literature has demonstrated. In ‘*Pedagogy of the Oppressed*’, Freire hinted that one of the prerequisites for actively decolonising education is for educators to possess what he refers to as a critical consciousness, which will allow them to assist their students in acquiring this crucial quality. Particularly in nursing education, the numerous effects of colonisation could be contested using Freire’s liberating pedagogy. The fundamental system that generates oppression could be challenged in nursing education by using the ideas of Freire’s *Pedagogy of the Oppressed* (1968). Non-Western ideas and views are seen to be irrelevant in nursing education and training because of the historical legacy of colonisation, which led to the white supremacy and whiteness of nursing curricula (Iheduru-Anderson & Waite 2024). White supremacy perpetuates white domination and non-white subordination in nursing education through pervasive conscious and unconscious attitudes in white superiority and entitlement (Waite & Nardi 2021).

Addressing the differences in the lived realities of racialised people and admitting unrecognised harm in all areas of professional nursing are essential components of the pedagogy for combating white supremacy. Additionally, it entails combating the ingrained white supremacist ideology that permeates nursing practice, academia, and experiential learning (Frediani 2020). Critical consciousness, conversation, humanisation, problem-posing education vs. banking education, praxis, oppression, and liberation are the main ideas of Freire’s ‘*Pedagogy of the Oppressed*’ (Freire 1968). The idea of critical consciousness is the focus of this article. According to Freire, education can empower underprivileged groups and advance social justice. He also emphasised the importance of critical consciousness in decolonising education and critical pedagogy. ‘Achieving a deep, meaningful, realistic, and reality-based understanding of one’s world’ is what is meant by ‘critical consciousness’. This involves seeing how those in positions of authority, wealth, and influence have brainwashed and conditioned those with perceived less power to think a certain way (Patton 2017). It entails critically analysing how society’s power and resources are distributed and acting to confront the status quo to fight oppression and advance equity. It teaches people to stand up to the oppressive aspects of their reality and to recognise the social, political, and economic contradictions. Through critical intercultural conversation and system reform, this attribute will encourage one to act to raise awareness and take strong critical stands against social injustices, particularly in nursing education (Patton 2017).

There are three stages of conscientisation as enumerated by Patton (2017): Magical, naïve, and critical awareness. When people have magical awareness, they use forces and powers outside of their comprehension and control to explain the events that have shaped their life. In contrast, people in naïve awareness accept principles, rules, and the social order they are in while not passively accepting their circumstances. However, they still do not fully comprehend the circumstances impacting their lives. Finally, in critical awareness or consciousness, people begin to examine their lived reality more closely and begin to challenge the norms, values, and expectations that have been instilled in them by those in positions of oppression, authority, and control. Thus, it is the nurse educator’s responsibility to thoroughly conscientise their students by guiding them through these stages. This calls for intentional, direct and proactive approach to addressing, exposing, and transforming instances of student nurses’ marginalisation by nursing academia. To stop racism in nursing education from continuing, they should recognise and confront racist viewpoints and behaviours that nursing students are socialised into (Neville et al. 2021).

By practising critical conscientiousness, nurse educators can confront power structures, become conscious of their own presumptions, and take action to create an inclusive and socially just learning environment that encourages students to develop critical consciousness (Ćumura & Petrović 2022). Critical consciousness, a key component of problem-posing education, encourages decolonisation and empowers nurse educators and students to critically examine historical and social contexts to build a democratic society in the profession. In nursing education, critical consciousness will enhance an understanding of social reality and their ability to design educational activities that bring to the fore thought-provoking conversations, enabling students to critically analyse the underlying causes of social injustices, inequities, and structural obstacles to equitable education (Ćumura & Petrović 2022).

To decolonise nursing education, the curriculum and teaching strategies must be critically examined and changed. Additionally, colonial legacies must be addressed, cultural humility must be encouraged, and equitable representation, inclusion, and respect for different viewpoints must be ensured (Waite & Nardi 2019). The goal of teaching for critical consciousness is to expose oppressive thought and behaviour patterns and strive for their eradication (Seider, Clark & Graves 2020). By participating in discussions that denounce banking education as ‘an instrument of oppression’, nursing scholars can act as agents of anti-oppressive pedagogy (Valderama-Wallace & Apesoa-Varano 2020). It is the responsibility of nurse educators to establish a learning environment that fosters the growth of critical consciousness in their students. This involves a component of ethical humility on the side of the nurse educator that helps to raise academic citizens’ critical awareness of the injustices experienced by people who are hurt by the current nursing education systems. In addition, those who continue to fight or persist to collaborate with those capable of fortifying what was rendered vulnerable, in the face of the urge to perpetuate colonial curriculum, must be encouraged (Belluigi 2023). In the end, critical consciousness will influence and motivate action, including what McDowell and Lock refer to as immersive learning experiences. According to Lock and McDowell (2023) immersion experiences can help develop contextual awareness and offer a critical consciousness dimension to learning that leads to personal development. To this end, Pillen, McNaughton and Ward (2020) suggest a framework for cultivating students’ critical consciousness that consists of introspection, information-creating disequilibrium, critical reflection priming, frame-of-reference revision, agency development for change, and combating oppression.

Finally, a decolonised curriculum, a critique of contemporary inequities in nursing education, and contemplation and action (praxis) to change the future are some of the ways that critical pedagogy education gives hope against the seemingly insurmountable job of achieving equity. Addressing inequalities involves more than just encouraging critical consciousness in ourselves as nurse educators and students. In the end, as nurse educators, we must have the courage to question the status quo and actively and aggressively modify conventional nursing education paradigms as part of our ‘social justice action’ or advocacy. We may advance critical consciousness and a decolonised nursing education by taking action in our individual and communal practice as educators. Furthermore, we are empowered to establish safe spaces and supportive learning environments that support the goal of decolonising the nursing curriculum. However, nurse educators are called upon to liberate themselves from the shackles of colonised minds and colonial nursing education and training.
